# Biorefining spent substrates of shiitake (*Lentinula edodes*) and oyster mushroom (*Pleurotus ostreatus*): enzymatic saccharification of cellulose and xylan, with lignin recovery from residues

**DOI:** 10.1080/21655979.2025.2536443

**Published:** 2025-07-30

**Authors:** Sarah J. Klausen, Luis A. Romero-Soto, Ayesha Liaqat, Zahra Dehghanmanshadi, Knut O. Strætkvern, Shaojun Xiong, Carlos Martín

**Affiliations:** a Department of Biotechnology, University of Inland Norway, Hamar, Norway; b Universidad Privada Boliviana, Facultad de Ingeniería y Arquitectura, La Paz, Bolivia; c Department of Forest Biomaterials and Technology, Swedish University of Agricultural Sciences, Umeå, Sweden; d Department of Chemistry, Umeå University, Umeå, Sweden

**Keywords:** Biorefinery, cellulose, enzymatic saccharification, biological pretreatment, lignin, oyster mushrooms, shiitake, spent-mushroom substrate

## Abstract

Spent mushroom substrate (SMS), the main by-product of mushroom cultivation, is a source of sugars that can be released by saccharification. This work aimed at investigating the enzymatic saccharification of the polysaccharides of the SMS of shiitake (*Lentinula edodes*) and oyster mushroom (*Pleurotus ostreatus*) and exploring the lignin extraction from the saccharification residues. First, analytical enzymatic saccharification (AES) with a cellulase cocktail and an experimental hemicellulase-rich preparation was applied. AES revealed higher digestibility of both polysaccharides for shiitake SMS than for oyster mushroom SMS. Using the cellulase cocktail, shiitake SMS resulted in a digestibility above 80% and 70% (w/w) for cellulose and xylan, respectively, while the maximum values for oyster mushroom SMS were 52% and 32% (w/w). The experimental enzyme preparation resulted in lower cellulose digestibility and higher xylan digestibility. Still, the saccharification trend between the two SMS types remained unchanged. To enhance the enzymatic saccharification of oyster mushroom SMS, hydrothermal treatment was applied. The treatment improved the enzymatic digestibility of cellulose by up to 84%. A validation experiment at larger scale showed that hydrothermally treated oyster mushroom SMS had a comparable overall conversion with non-treated shiitake SMS. Following a biorefinery strategy, lignin was extracted from the residues of the preparative enzymatic saccharification using the green solvent γ-valerolactone under different temperatures and holding times. The extracted product contained 98.8% lignin and did not contain cellulose or xylan. The results of this study provide the grounds for biorefinery processes enabling recovery of bioactive compounds, fermentable sugars, and high-quality lignin from SMS

## 1. Introduction

Solid-state fermentation of residual plant biomass to produce edible mushrooms is a comprehensive approach to valorizing unexploited agricultural and forest residues [1]. Mushroom cultivation is a sustainable process that addresses waste management and provides a means of producing food sources. Furthermore, since mushroom production is not affected by weather and seasonality, it contributes to food security and rural development [2].

Mushrooms are cultivated on lignocellulosic substrates, i.e. materials composed of lignin, cellulose, and hemicelluloses. Cereal bran, legume flour, and mineral salts are added to the substrate to provide the required nutrients and ensure reasonably rapid growth and a high mushroom yield [3]. During the cultivation, the fungal enzymes degrade the substrate components to release nutrients, promoting fungal growth. The mushroom cultivation process concludes with harvesting the fungal fruitbodies, leaving behind a residual material known as spent mushroom substrate (SMS). Depending on the species and the number of harvest flushes, around 3–5 kg of SMS is generated per kg of produced mushrooms [4]. The SMS is the main by-product of the mushroom industry, and its generation is increasing steadily due to the rapid growth of the mushroom market [5]. The large amounts of generated SMS are a significant economic challenge for mushroom producers, and the disposal by incineration, landfilling, or spreading on land causes environmental pollution [6]. Although SMS is currently perceived as a waste with little value, it holds potential for different applications, contributing to transitioning to a circular economy model [7].

A possible direction for SMS valorization is its use as biorefinery feedstock, where different constituents are separated sequentially and directed to appropriate applications [8]. Although the substrate polymers, i.e. cellulose, hemicelluloses, and lignin, are partially consumed by the fungi during cultivation, a considerable fraction of them remain undigested in the SMS and holds an immense value for biorefinery applications [9]. SMS can be considered a source of sugars that can be released by enzymatic saccharification of cellulose and hemicelluloses and then used in microbial fermentations to produce biofuels, biopolymers, or platform chemicals [10]. As a consequence of the saccharification of the SMS polysaccharides, a lignin-rich solid residue is generated. Lignin extraction from the residue can facilitate the recovery of a valuable feedstock for producing bio-carbon materials, composites, and nanoparticles substituting fossil-based products [11]. Furthermore, SMS contains bioactive compounds from the fungal mycelium and substrate phytochemicals, which are also valuable for biorefinery processing [12]. Bioactive compounds extracted from SMS can be directed to biomedical, nutraceutical, and pharmaceutical applications.

An important feature of SMS cellulose as a substrate for enzymatic saccharification is because of its enhanced susceptibility toward enzymes. The partial consumption of lignin and hemicelluloses during cultivation contributes to removing barriers that commonly affect the enzymatic saccharification of lignocellulosic biomass [13]. Therefore, mushroom cultivation can *per se* be considered a biological pretreatment for improving the enzymatic digestibility of cellulose and preparing the lignocellulosic materials for further bioconversion [14]. As a biological pretreatment, mushroom cultivation holds advantages over chemical and thermo-chemical pretreatment methods [10] regarding low-temperature operation, fewer added chemicals, and low formation of fermentation inhibitors [15].

Biological pretreatment using lignin-degrading fungi has been previously proposed as a low-cost alternative to treat lignocellulosic biomass prior to enzymatic saccharification [16]. However, its slow conversion rate makes it non-viable as a stand-alone method. Combining the cultivation of edible fungi with subsequent enzymatic saccharification opens new perspectives since the co-production of food and fermentable sugars allows for increasing the cost-effectiveness of biological pretreatment [17].

Shiitake (*Lentinula edodes*) and oyster mushrooms (*Pleurotus* spp.) are among the top-consumed edible mushrooms worldwide [18]. Using them in biological pretreatment for the combined production of food and fermentable sugars can have a huge economic and social impact. This group has previously shown the effectiveness of shiitake cultivation as a biological pretreatment, resulting in high cellulose conversion [9,10,14,19]. Some examples show that oyster mushroom cultivation led to spent substrates with enhanced saccharification efficiency [17,20]. However, there are no reports comparing the effectiveness of shiitake and oyster mushrooms in enhancing the enzymatic saccharification of cellulose. Furthermore, the studies on enzymatic saccharification of polysaccharides contained in SMS from either shiitake or oyster mushrooms are generally focused on cellulose, while the saccharification of hemicelluloses is overlooked.

The recovery and valorization of lignin contained in the residues of the enzymatic saccharification of SMS is another aspect that has not received enough attention. The few existing reports deal only with lignin extraction from raw SMS [21,22], while lignin recovery from SMS saccharification residues has been ignored so far.

The current study thoroughly investigates the enzymatic saccharification of cellulose and xylan contained in spent substrates from cultivating shiitake and oyster mushrooms. This allows the gathering of experimental data for a comparative assessment of the effectiveness of both fungal species as biological pretreatment agents for lignocellulosic biomass. Additionally, this study presents a first approach to extracting lignin from SMS saccharification residues.

## 2. Materials and methods

### 2.1. Materials

Spent mushroom substrate (SMS) from the cultivation of shiitake and oyster mushrooms was used. The oyster mushroom SMS, provided by Husbonden AS (Disenå, Norway), corresponded to a *Pleurotus ostreatus* × *Pleurotus eryngii* hybrid strain cultivated on an initial substrate containing 60% (w/w) lignocellulose and 40% water. The lignocellulosic part of the substrate consisted of oak sawdust and wheat bran, representing 80% and 20% (w/w) of the dry weight. The initial substrate also contained 0.08% (w/w) of CaCO_3_. The shiitake (*Lentinula edodes*) SMS, provided by the Swedish University of Agricultural Sciences (Umeå, Sweden), was collected after the cultivation on an initial substrate based on birch sawdust (80%) and wheat bran (20%). The SMS samples were dried at room temperature until a dry-matter (DM) content above 90% (w/w) was reached. The particle size of the dry samples was reduced by using a Robot Coupe R3 shredder with a serrated blade (Robot-Coupe SNC, Montceau-en-Bourgogne, France). The shredded material was then screened, and the fraction with particle size below 0.5 mm was collected and stored in sealable plastic bags until further use in enzymatic saccharification, extractives removal, and compositional analysis.

The enzyme preparation Cellic CTec2, commercialized as *Cellulase, enzyme blend* by Sigma-Aldrich (Steinheim, Germany), was used for enzymatic saccharification. Cellic CTec2 consists of cellulases, β-glucosidase, other cellulose-degrading enzymes, and hemicellulases. A hemicellulases-rich raw experimental cocktail (ExpC) supplied by a major enzyme producer under MTA conditions was also used. The total cellulase activity of the enzyme preparations was measured as filter paper activity (FPA) following Ghose’s protocol [23]. The xylanase activity was determined by quantifying the released xylose from beechwood xylan according to the method developed by Ghose and Bisaria [24]. The Cellic CTec2 displayed 148 filter paper units (FPU) and 1.4 xylanase kU per mL. The activities of the ExpC preparation were 71 FPU/mL and 141 xylanase kU/mL. The enzyme preparation Cellic CTec3 HS, used in the preparative enzymatic saccharification, was donated by Novozymes A/S (Bagsværd, Denmark).

All the chemicals used in this study were obtained from Sigma-Aldrich Chemie GmbH (Steinheim, Germany) and Merck (Darmstadt, Germany).

### 2.2. Removal of extractive compounds

Before enzymatic saccharification, in some SMS samples, the extractive compounds were removed by sequential extraction using distilled water and 96% ethanol as solvents in a Soxhlet apparatus. SMS samples (10 g) were placed in a Cytiva’s Whatman cotton cellulose thimble (Alundum thimbles, China), which was then inserted into the extraction chamber of the Soxhlet apparatus and extracted for eight hours with 250 mL of each solvent. The moisture contained in the SMS sample was considered in calculating the volume of the liquid phase. When the extraction was completed, the extracts were preserved at 5°C in the refrigerator, while the extract-free solids were air-dried until constant weight and then used in enzymatic saccharification experiments.

### 2.3. Enzymatic saccharification

#### 2.3.1. Analytical enzymatic saccharification

Following our recently described protocol, analytical enzymatic saccharification was performed at 5% (w/w) solids load [25]. Around 50 mg (DW) of SMS were suspended in 900 μL of 50 mM sodium citrate buffer (pH 5.2) in 1.5-mL Eppendorf tubes. The tubes with the suspensions were mixed by vortexing at room temperature and placed in an incubator (Termaks B4115, Bergen, Norway) for one hour at 45°C. After that, 50 μL of a previously prepared stock solution containing the exact volume of the enzyme, either Cellic CTec2 or ExpC, was added to achieve the dosage required in each experiment (15 or 25 FPU/g biomass). After that, the saccharification was run for 72 h. By the end of the hydrolysis, the tubes were centrifuged at 20,000 g for 5 min. The supernatant was recovered, and the solid residue was discarded. A portion of the supernatant was used for sugar analysis by HPLC, and the rest was stored frozen. The glucose and xylose values quantified by HPLC were used for calculating the enzymatic digestibility of cellulose and xylan.

(1) $$\mathrm{Enzymaticdigestibilit}y_{\mathrm{Cellulose}}=\frac{\mathrm{Cel}l_{\mathrm{EH}}}{\mathrm{Cel}l_{\mathrm{SMS}}}\times 100$$

(2) $$\mathrm{Enzymaticdigestibilit}y_{\mathrm{Xylan}}=\frac{\mathrm{Xy}l_{\mathrm{EH}}}{\mathrm{Xy}l_{\mathrm{SMS}}}\times 100$$

*Cell*_*EH*_ and *Xyl*_*EH*_ are the masses (in grams) of saccharified cellulose and xylan, which are based on the concentrations of glucose and xylose in the enzymatic hydrolyzates. *Cell*_*SMS*_ and *Xyl*_*SMS*_ are the masses (in grams) of cellulose and xylan in the SMS sample used in the enzymatic saccharification assay.

#### 2.3.2. Preparative enzymatic saccharification

Preparative enzymatic saccharification was performed to validate the results of the analytical enzymatic saccharification at a larger scale. The experiment used raw shiitake SMS and hydrothermally treated oyster mushroom SMS at a 10% (w/w) substrate load as previously described [25]. Around 15 g (DW) of SMS suspended in 135 mL of 50 mM sodium citrate buffer (pH 5.2) was used in each experimental run. A state-of-the-art enzyme preparation (Cellic CTec3 HS) was used. The dosage of the enzyme preparation was based on the supplier’s indications. The saccharification was performed at 45°C and 170 rpm for 72 h in 500-mL Erlenmeyer flasks placed in a New Brunswick Innova 44 incubator shaker (Eppendorf, Hamburg, Germany). The flask capacity represented a 1:333 scale-up compared to the analytical enzymatic saccharification. Samples for sugar analysis were taken after 5, 10, 24, 32, 48, and 72 h. By the end of the saccharification time, the hydrolyzate was separated by centrifugation at 20,000 g for 15 minutes. The separation was completed by filtering the supernatants under vacuum. The sugar concentrations were quantified by HPLC, and the hydrolyzates were stored frozen for another study. The solid residues from saccharification were washed with abundant water and air dried until constant weight. The experiment was run in duplicates.

### 2.4. Hydrothermal treatment

Hydrothermal (HT) treatment of oyster mushroom SMS was performed by heating a mixture containing 30 g (DW) of SMS suspended in 270 mL of distilled water in a pressurized Parr 4520 reactor (Parr Instrument Company, Moline, IL, USA). The contribution of the SMS moisture was considered for calculating the water volume. As previously reported, two heating modes, non-isothermal and partially isothermal, were applied [25]. In the non-isothermal mode, the SMS suspension was treated non-isothermally by heating it to 175°C and cooling it to room temperature immediately afterward. In the partially isothermal mode, the suspension was heated to 175°C and held at that temperature for 30 min before cooling it down. Cooling to room temperature was performed by passing cold water through an internal coil controlled by a solenoid valve module. After reaching 30°C, the reactor was emptied, and the slurry was submitted to vacuum filtration to separate the treated solids from the liquor. The pretreated solids were washed with distilled water, then air-dried until a DM content above 90% (w/w), and stored at room temperature until further analysis and enzymatic saccharification. Two criteria, namely the enzymatic digestibility of treated cellulose (EDPC) and overall enzymatic conversion (OEC), were used to assess the saccharification of the pretreated solids. The EDPC was based on the cellulose and xylan contained in the pretreated solids. In contrast, the OEC was based on the cellulose and xylan contained in the SMS before the HT treatment.

### 2.5. Lignin extraction from the saccharification residues

Lignin was extracted from the residues of the enzymatic saccharification of shiitake SMS using a solvent system consisting of γ-valerolactone (GVL) and distilled water in an 80:20 volume ratio based on previous experience with other biomass materials [26]. Seven extraction runs at temperatures ranging from 120°C to 150°C and 20–40 min holding times were performed in a Parr 4520 reactor. The experiment included triplicate runs at 135°C for 30 min. A 19:1 liquid-to-solid ratio (15 g (DW) of SMS and 285 mL of the solvent mixture) was used in each experimental run. After the extraction time had elapsed, the liquor containing solubilized lignin was separated from the solid residue by vacuum filtration. The filter cake was washed first with a portion of the solvent mixture and then with distilled water, air-dried, and weighed to calculate the yield of treated solids. Lignin solubilization was determined by a material balance including the masses of the starting material and the resulting filter cakes and their respective lignin content.

Lignin was regenerated from the liquors by precipitation using water as an anti-solvent. Three volumes of water were added to the liquors, and the liquid mixture was then refrigerated at 4°C overnight. After that, the precipitated lignin was separated by vacuum filtration and centrifugation at 13,000 g for 30 min at 4°C using a Heraeus Pico 21 centrifuge (Thermo Fisher Scientific, Osterode, Germany). The regenerated lignin was air-dried at room temperature for two-three days, weighed, and submitted to compositional analysis by analytical acid hydrolysis.

### 2.6. Analysis

The chemical composition of the SMS samples and saccharification residues was determined following standard methods. The content of extractive compounds was determined gravimetrically after sequential extraction with water and 96% ethanol in a Soxhlet apparatus [27]. The mineral content was determined as ash after incineration at 575°C for five hours in a Carbolite CWF 1100 muffle furnace (Carbolite Gero, Sheffield, UK) following the National Renewable Energy Laboratory (NREL) standard protocol [28]. The content of structural carbohydrates and lignin was determined by analytical acid hydrolysis, followed by HPLC quantification of the released sugars and gravimetric determination of lignin [29].

The concentrations of glucose, xylose and arabinose were quantified in all hydrolyzates by HPLC using an Ultimate 3000 (Dionex Softron, Germering, Germany) system. A Rezex RPM Monosaccharide Pb^2+^ column (Phenomenex, Torrance, CA, USA), maintained at 85°C, was used to analyze the hydrolyzates from analytical enzymatic saccharification. The eluent was distilled water with a flow rate of 0.6 mL/min. The hydrolyzates from analytical acid hydrolysis and preparative enzymatic saccharification were analyzed using an Aminex HPX-87 H column (Bio-Rad, Hercules, CA, USA) maintained at 65°C. The eluent was a 5 mM aqueous sulfuric acid solution supplied at a 0.6 mL/min flow rate. A refractive index (RI) detector (Showa Denko, Tokyo, Japan) was used in both methods. Before HPLC analysis, all the samples were appropriately diluted and filtered using 0.45 µm Nylon syringe filters (VWR, Radnor, PA, USA).

To assess statistical significance between datasets, a Student’s t-test was performed to compare the obtained results between both types of SMS. First, the mean and standard deviation were calculated for each component in both groups, then, hypotheses were set. Finally, the *p*-value was obtained for each component and compared to a significance level (α = 0.05) to determine whether the differences observed were statistically significant.

## 3. Results and discussion

**Figure 1.**
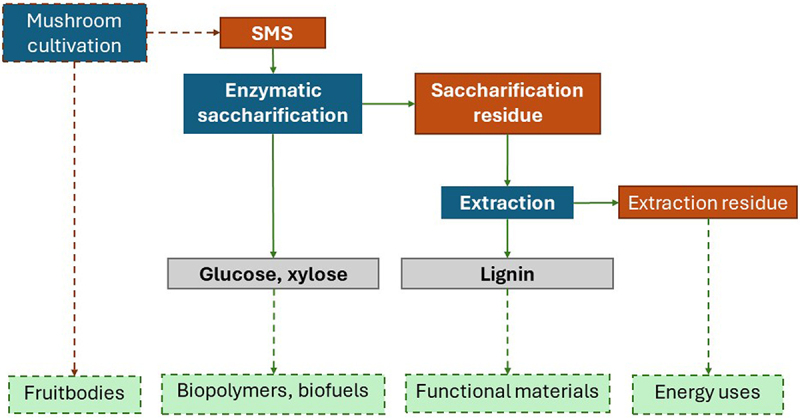
Schematic representation of the experimental flow. The dashed lines and squares represent processes or products that are beyond the scope of this article but within the proposed biorefinery approach.

The schematic layout of the biorefinery approach followed in this study is presented in Figure 1. The spent mushroom substrate (SMS) from the cultivation of shiitake and oyster mushrooms received from the mushroom producers was submitted to enzymatic saccharification to produce sugars, i.e. glucose and xylose. The enzymatic saccharification also resulted in a solid residue that was then subjected to extraction to recover lignin. The sugars and lignin, as well as the extraction residues, are directed to other uses beyond the scope of this study.

### 3.1. Characterization of the spent mushroom substrate

Characterization of the chemical composition of the SMS was performed before starting with the saccharification experiments. The results are given in Table 1. For both SMS types, the polysaccharides cellulose and xylan were the main structural constituents, representing around 39–47% (w/w) of the total mass. This shows the potential of SMS as a source of sugars to be made available by enzymatic saccharification of the polysaccharides.

**Table 1.** Composition (% w/w) of the spent mushroom substrates of shiitake and oyster mushroom. Mean values and standard deviations from triplicate measurements (*n* = 3).

Component	Shiitake SMS	Oyster mushroom SMS
Cellulose^1^	28.1^a^ ± 0.3	30.6^a^ ± 1.7
Xylan	10.6^a^ ± 0.3	15.8^b^ ± 0.8
Arabinan	2.2^a^ ± 0.3	2.8^b^ ± 0.3
Lignin^2^	6.6^a^ ± 0.3	12.7^b^ ± <0.1
Water extractives	22.3^a^ ± <0.1	11.6^b^ ± 0.2
Ethanol extractives	2.8^a^ ± 0.4	1.4^b^ ± 0.3
Ash	5.4^a^ ± 0.8	2.6 ^b^ ± <0.1

^1^Determined as glucan; ^2^Determined as Klason lignin; For each component, different superscript letters (^a,b^) indicate statistically significant difference between the two SMS; Similar superscript letter (^a,a^) stand for no significant difference.

Oyster mushroom SMS had a higher content of carbohydrates and lignin than shiitake SMS (Table 1). The content of cellulose, the main SMS component, was 28.1 ± 0.3% (w/w) for shiitake and 30.6 ± 1.7% (w/w) for oyster mushroom. The differences between the cellulose content values in both SMS were not statistically significant (*p* = 0.07) at a 95% confidence level. However, significant differences were found between the content of xylan (*p* < 0.05) and lignin (*p* < 0.05) in both materials. Lignin content was around two-fold higher in oyster SMS (12.7% (w/w)) than in shiitake SMS (6.6% (w/w)), while xylan content was ~ 50% higher (15.8% (w/w) vs 10.6% (w/w)).

The lignocellulosic composition of the SMS is largely a consequence of the degradation of the main components of the substrate during fungal growth. Chen et al. [17,19] showed that the changes in substrate lignocellulose during the cultivation were mainly attributed to the cultivated fungal species rather than the tree species used as initial substrate. The degradation pattern observed for shiitake corroborates some of our previous observations [14,19] and agrees with other reports on compositional changes during shiitake cultivation [30]. The higher content of xylan and lignin in oyster mushroom SMS than in shiitake SMS might be related to lower degradation due to a shorter cultivation period. Oyster mushroom cultivation can be completed in around 6–7 weeks [31], while in shiitake cultivation, harvesting the first flush takes around 8–10 weeks [30].

The content of extractive compounds was high for both SMS (Table 1). For shiitake SMS, water extractives represented the second highest share (22.3% (w/w)) of the compositional analysis. The share of water extractives was lower for oyster mushroom SMS than for shiitake SMS but still relatively high (11.6% (w/w)). The high content of extractives can be attributed to metabolites from fungal cultivation and the formation of water- and ethanol-soluble degradation products during fungal degradation of polysaccharides and lignin. The higher content of extractives in shiitake SMS compared with oyster mushroom SMS, as the lower content of polysaccharides and lignin, can be consequences of a longer cultivation process for shiitake than for oyster mushroom. Shiitake SMS contained more ash than oyster mushroom SMS (Table 1). The different ash content in both materials might be attributed to the initial substrates used for cultivation. The content of extractives and ash aligns with previous reports for both SMS. Klausen et al. [12] reported comparable levels of water extractives (13.7%), ethanol extractives (1.3%), and ash (2.6%) for SMS derived from the cultivation of oyster mushroom on an initial substrate composed of oak sawdust and wheat bran. Chen et al. [19] reported comparable content of extractives and ash for SMS derived from the cultivation of shiitake grown on birch sawdust and wheat bran.

### 3.2. Enzymatic saccharification of oyster mushroom SMS

**Figure 2.**
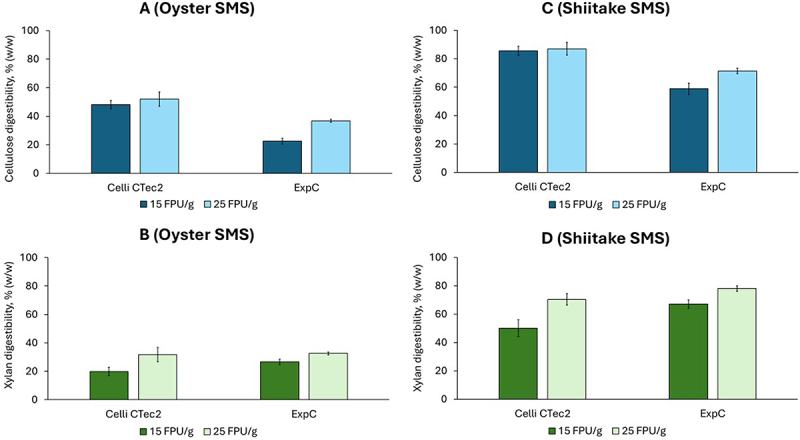
Enzymatic digestibility of cellulose (A, C) and xylan (B, D) contained in oyster mushroom SMS (left-side panels) and shiitake SMS (right-side panels) using the Cellic CTec2 and ExpC enzyme preparations at 15 and 25 FPU/g. The error bars show the standard deviations of means (*n* = 3).

The saccharification of cellulose and xylan was evaluated with two enzyme preparations at two different dosages and following an analytical enzymatic saccharification protocol, which is a useful approach to evaluate a series of feedstock samples, pretreatment methods, or enzyme preparations [32]. The enzymatic digestibility, expressed as the mass percentage of cellulose or xylan saccharified during the assay, was the criterion for assessing the saccharification efficiency. The enzymatic digestibility of cellulose and xylan during the enzymatic saccharification of oyster mushroom SMS is shown in Figure 2 (panels A and B).

The enzymatic digestibility of cellulose was better with the commercial preparation Cellic CTec2 than with the experimental cocktail ExpC (Figure 2A). For both preparations, the digestibility increased with increasing the enzyme dosage. The saccharification with Cellic CTec2 resulted in cellulose digestibility values of 48.1% and 52.0% (w/w) for 15 and 25 FPU/g dosages, respectively (Figure 2A). When the ExpC preparation was used, the cellulose digestibility was lower, with a maximum of only 36.8% (w/w) at the highest enzyme dosage. Xylan digestibility was slightly better for ExpC (26.6 ± 2.0% (w/w)) than for Cellic CTec2 (19.9 ± 2.7%) when a low enzyme dosage (15 FPU/g) was used (Figure 2B). However, at higher enzyme dosage (25 FPU/g), xylan digestibility was comparable for ExpC (32.6 ± 0.7% (w/w)) and for Cellic CTec2 (31.7 ± 3.5% (w/w)).

### 3.3. Enzymatic saccharification of shiitake SMS

The results of the enzymatic saccharification of cellulose and xylan contained in shiitake SMS are presented in Figure 2 (panels C and D). The enzymatic saccharification of shiitake SMS resulted in maximum cellulose digestibility values of 87.0% and 71.4% (w/w)) using the enzyme preparations Cellic CTec2 and ExpC, respectively (Figure 2C). For Cellic CTec2, the cellulose digestibility (85.6 ± 3.2 and 87.0 ± 4.5) was comparable for both enzyme dosages assayed, whereas an apparent increase from 58.9% at 15 FPU/g to 71.4% at 25 FPU/g was observed for the ExpC preparation. Even though similar cellulase dosages, measured as FPU/g, were used for both enzyme preparations, Cellic CTec2 outperformed ExpC regarding cellulose digestibility. The reason might be that β-glucosidase or other activities required for cellulose saccharification are lacking in ExpC, while they are found in Cellic CTec2. β-Glucosidase activity and lytic polysaccharide monooxygenases (LPMOs) in Cellic CTec2 are well documented [33,34]. However, no data on the presence of those activities in ExpC are available.

For Cellic CTec2, the highest enzymatic digestibility of xylan was 70.3% (w/w) (Figure 2D), which was lower than the value achieved for cellulose. A different trend was observed for the saccharification using the ExpC preparation, which resulted in higher digestibility of xylan (78.0% (w/w)) than that of cellulose (71.4% (w/w), Fig. 2C). For both preparations, higher enzyme dosages resulted in higher xylan digestibility. The better xylan digestibility observed for ExpC is well correlated to the higher xylanase activity of this preparation.

For both cellulose and xylan, the enzymatic saccharification of shiitake SMS yielded higher digestibility values than those achieved for oyster mushroom SMS. This trend might be linked to the lignin content, which was lower for shiitake SMS (6.6% (w/w)) than for oyster mushroom SMS (12.7% (w/w) (Table 1). It is known that the enzymatic saccharification of cellulose is inhibited by the adsorption of cellulases to lignin [35] and that a decreased lignin content in lignocellulosic biomass leads to improved saccharification.

The better enzymatic saccharification observed for shiitake SMS than that achieved for oyster mushroom SMS indicates that shiitake cultivation is a better pretreatment method than oyster mushroom cultivation to facilitate enzymatic saccharification. Therefore, shiitake cultivation can be applied to lignocellulosic biomass as a biological pretreatment method, allowing a high yield of sugars upon enzymatic saccharification. This group has previously shown that shiitake cultivation is an efficient pretreatment method prior to the enzymatic saccharification of cellulose [14,19], but this is the first report where the effectiveness of shiitake and oyster mushroom cultivation as pretreatment methods are compared for both cellulose and xylan.

### 3.4. Comparison of the enzymatic saccharification in raw and extract-free SMS

The extraction of bioactive compounds is a valorization alternative for SMS [12]. The extract-free material resulting from the extraction of bioactive compounds is rich in polysaccharides that are of interest as sugar sources. To investigate how the extraction affects the susceptibility of cellulose and xylan to saccharifying enzymes, the enzymatic saccharification of raw and extract-free SMS was evaluated. Cellic CTec2 was used, considering it was the best-performing enzyme preparation in the previous experiments. A dosage of 15 FPU/g was chosen because of economic reasons and considering that cellulose digestibility for shiitake SMS resulted in comparably high values independently of the used enzyme dosage (Figure 2C).

**Figure 3.**
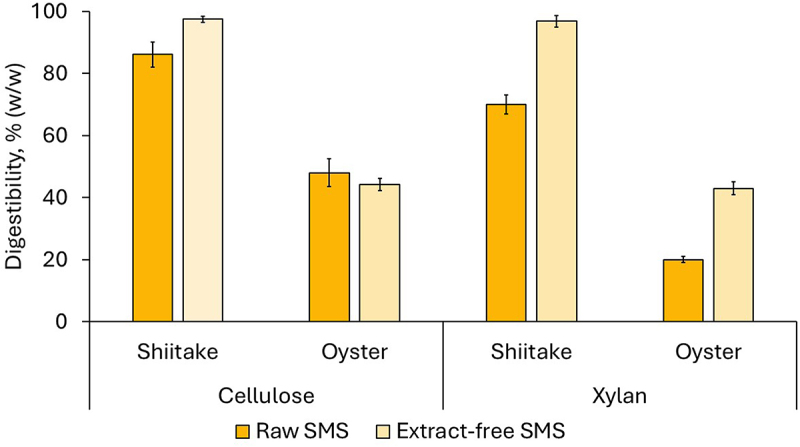
Comparison of the enzymatic digestibility of cellulose and xylan during saccharification of raw and extract-free SMS from shiitake and oyster mushroom. Cellic CTec2 was used at a 15 FPU/g dosage. The error bars show the standard deviations of means (*n* = 3).

The experiment revealed, in general, an improvement in the enzymatic digestibility of both polysaccharides when the extractive compounds were removed (Figure 3). The improvement was clearly detectable for xylan in both SMS types and for cellulose in shiitake SMS. Still, it was not evident for cellulose in oyster mushroom SMS, where the digestibility was 48.0 ± 4.5% for the raw SMS and 44.3 ± 2.0% for the extract-free one.

The positive effect of the extraction on the enzymatic saccharification agrees with a previous study by Klausen et al. [12]. The improvement of the enzymatic saccharification after extraction can be attributed to the removal of certain extractive compounds that might have an inhibitory effect on the cellulases [15]. However, the lack of improvement for oyster mushroom SMS indicates that, probably due to the different nature of the extracted compounds, the effect does not apply to all SMS types. Even though, enhancing the enzymatic digestibility of SMS polysaccharides after removal of the extractive compounds opens the possibilities for a cascade processing of SMS for recovering bioactive compounds as the first step and producing sugars as the second step.

### 3.5. Hydrothermal (HT) treatment of oyster mushroom SMS

HT treatment was conducted on oyster mushroom SMS because its enzymatic digestibility was lower compared to that of shiitake SMS (Figure 2). The aim of the HT treatment was to enhance the susceptibility of oyster mushroom SMS to the enzymes so that an enzymatic digestibility comparable to that of shiitake SMS could be achieved.

#### 3.5.1. Effect of the HT treatment on the composition and recovery of the lignocellulose polymers

**Figure 4.**
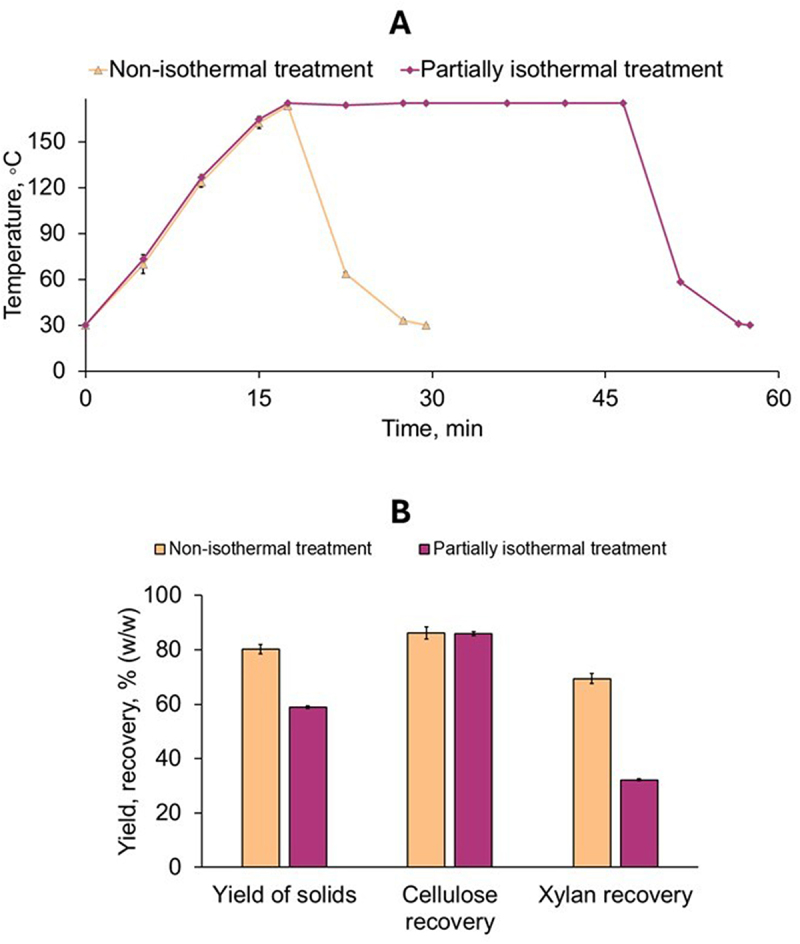
Hydrothermal (HT) treatment of oyster mushroom SMS under non-isothermal (amber line and columns) and partially isothermal (violet line and columns) heating modes. A, temperature profile of the treatment; B, yield of solids and recovery of cellulose and xylan after treatment. The error bars show the standard deviations of means (*n* = 2).

The HT treatment was performed under either non-isothermal or partially isothermal heating mode with 175°C as the temperature setpoint. In the non-isothermal heating mode, the SMS suspension was heated to 175°C and cooled down immediately. In the partially isothermal mode, the temperature setpoint was held constant for 30 minutes (Figure 4A).

The hydrothermal treatment resulted in a solids’ yield of 80.2% (w/w) for the material treated under the non-isothermal heating mode and 58.9% (w/w) for the material from the treatment under partially isothermal mode (Figure 4B), which showed a significant difference between the two treatments (*p* < 0.01). The cellulose content increased from 30.4% (w/w) in the raw SMS to 31.6% (w/w) in the treated solids from the HT treatment under non-isothermal mode and to 42.5% (w/w) in those from the partially isothermal mode (Table 2). Xylan content decreased from 14.7% (w/w) in the raw SMS to 12.3% and 7.7% (w/w), respectively, in the solids from the treatments under non-isothermal and partially isothermal heating modes. Arabinan was not detected in any of the treated solids. The lignin content was 14.7% (w/w) in the raw SMS and increased to 25.4% (w/w) in the treated solids from the non-isothermal mode and 35.7% (w/w) in those from the partially isothermal mode.

**Table 2.** Composition (% w/w) of the spent substrate of oyster mushroom before and after hydrothermal treatment under non-isothermal and partially isothermal heating modes. Mean values and standard deviations from triplicate measurements.

Component	Raw SMS^1^	Treated solids from the non-isothermal mode	Treated solids from the partially isothermal mode
Cellulose^2^	30.4 ± 0.6	31.6 ± <0.1	42.5 ± 0.9
Xylan	14.7 ± 0.3	12.3 ± <0.1	7.7 ± 0.3
Arabinan	1.1 ± <0.1	ND	ND
Lignin^3^	15.3 ± 1.5	25.4 ± 1.1	35.7 ± 1.2

^1^SMS before pretreatment; ^2^Determined as glucan; ^3^Determined as Klason lignin; ND, not detected.

The decreased solids yield after HT treatment (Figure 4B) can be attributed to the solubilization of hemicelluloses and extractives. The solubilization of hemicelluloses during the treatments was revealed by mass balances. The balances showed a xylan recovery of 69.4% (w/w) in the treatment under non-isothermal heating mode (Figure 4B). This indicates that around one-third of the initial xylan was solubilized in that treatment. The solubilization was two-fold higher (*p* < 0.01) in the treatment under partially isothermal mode, where xylan recovery was only 32.2% (w/w), corresponding to a solubilization of around two-thirds (67.8%) of the initial xylan. The second hemicellulosic component, arabinan, was completely solubilized under both heating modes. Because of hemicelluloses’ solubilization, the treated solids were enriched in cellulose and lignin as shown by increased content after HT treatment. The cellulose and lignin content in the treated solids was higher for the partially isothermal mode than for the non-isothermal one, which agrees well with the solubilization trend observed for hemicelluloses. Some cellulose loss occurred during treatment, as indicated by the cellulose recovery below 100%. It is noteworthy that the cellulose recovery (83.2 ± 2.3% (w/w) and 81.6 ± 0.2% (w/w)) was comparable for both heating modes (*p* > 0.05).

#### 3.5.2. Analytical enzymatic saccharification of treated SMS

As a first step to evaluate the effect of the hydrothermal treatment on cellulose saccharification, an analytical enzymatic saccharification assay [32] was tested. The assay was performed with 1-mL suspensions of treated solids of oyster mushroom SMS at a low consistency (5% solids) in Eppendorf tubes using the enzyme preparation Cellic CTec2.

**Figure 5.**
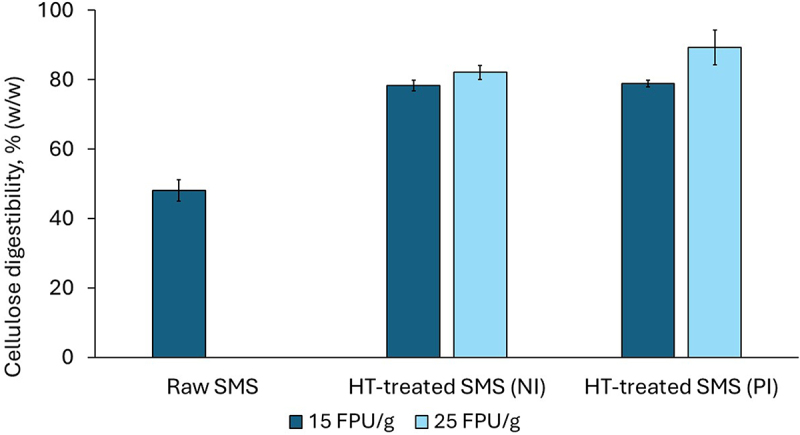
Enzymatic saccharification of cellulose in hydrothermally (HT) treated oyster SMS. Cellic CTec2 was used at 15 and 25 FPU/g dosages. Raw oyster SMS was used as a reference. NI and PI indicate non-isothermal and partially isothermal heating modes. The error bars show the standard deviations of means (*n* = 3).

The experiment showed that HT treatment under relatively mild conditions resulted in enhanced enzymatic saccharification of cellulose compared to the raw SMS (Figure 5). The enzymatic digestibility of cellulose for both treatments ranged between around 78.0% and 89.0% (w/w) (Figure 5). These values are approximately 62.0% − 84.0% higher than in the non-treated oyster SMS and are comparable with those of raw shiitake SMS (Figure 2C). The highest enzymatic digestibility (89.3 ± 5.0% (w/w)) was achieved for the treated solids from the partially isothermal treatment using the highest enzyme dosage (25 FPU/g). For the treated solids from the treatment performed under the partially isothermal mode, the enzymatic digestibility significantly increased (*p*-value <0.02) with the enzyme dosage. A less remarkable effect of the enzyme dosage was observed for the solids from the non-isothermal treatment, which yielded 78.3 ± 1.5% and 82.1 ± 2.1% (w/w) for 15 and 25 FPU/g, respectively. The enhanced enzymatic digestibility after HT treatment agrees with previous reports using hydrothermal processing as a pretreatment method for different types of lignocellulosic biomass [36].

### 3.6. Assessment of the enzymatic saccharification of SMS at preparative scale

After the small-scale analytical enzymatic saccharification revealed the effect of a mild HT treatment on the enzymatic digestibility of oyster mushroom SMS cellulose, it was decided to validate the results in a larger-scale experimental setup using a higher consistency of solids in the reacting suspension and with a state-of-the-art enzyme preparation. The solids load was twice as much (10% vs 5%) as that in the analytical enzymatic saccharification. The enzyme dosage recommended by the supplier was used. The experimental setup allowed us to elucidate the sugar release dynamics and compare the saccharification of hydrothermally treated oyster mushroom SMS with that of raw shiitake SMS, which was included in the experiment. The experimental approach is termed preparative enzymatic saccharification because it was also directed to produce hydrolyzates to be used in fermentation experiments (beyond the scope of the current study).

**Figure 6.**
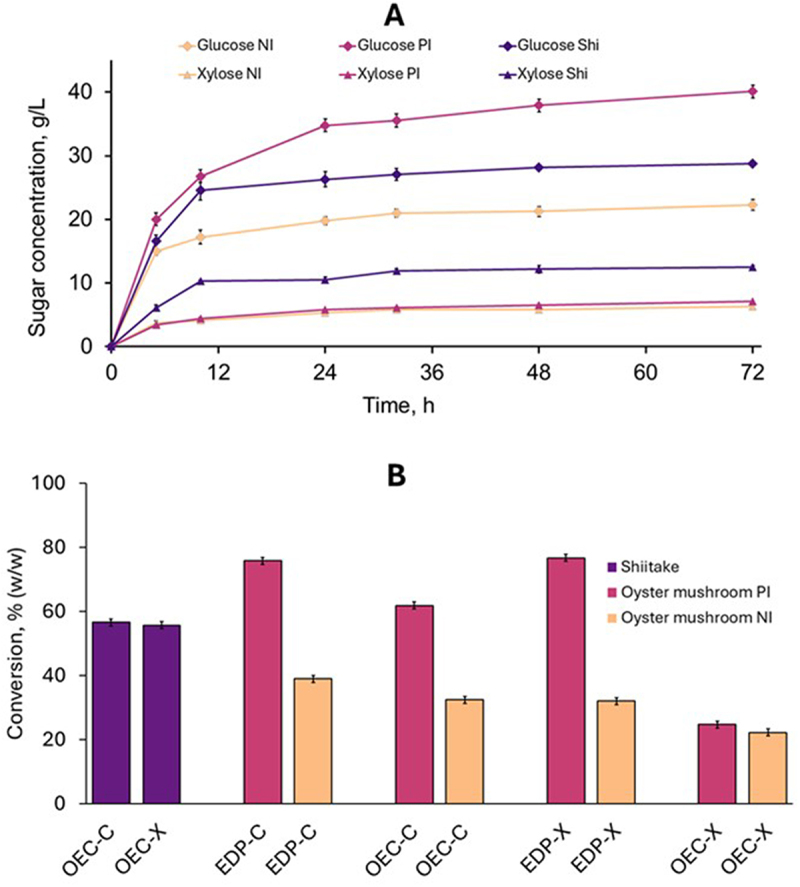
Preparative enzymatic saccharification of SMS. A, dynamics of the release of glucose (rhombs) and xylose (triangles) during saccharification of raw shiitake SMS (violet lines) and hydrothermally treated oyster mushroom SMS under non-isothermal (amber lines) and partially isothermal (purple lines) heating. B, enzymatic digestibility of treated polysaccharides (EDP-) and overall enzymatic conversion (OEC-). The C and X at the end of the coding stand for cellulose and xylan, respectively. The error bars show the standard deviations of means (*n* = 2).

The release of glucose and xylose, evident for all the materials already after 5 h of saccharification (Figure 6A), was monitored during the process. The oyster mushroom SMS treated under partially isothermal heating resulted in the highest glucose concentration (40.1 ± 0.61 g/L). In comparison, the sample treated under non-isothermal heating mode resulted in lower glucose formation (22.3 ± 0.01 g/L) (*p* < 0.01). Shiitake SMS yielded a glucose concentration of 28.8 ± 0.86 g/L (Figure 6A), an intermediate value between the results achieved for the two hydrothermally treated oyster mushroom SMS samples (*p* < 0.01). It should be kept in mind that shiitake SMS had not been subjected to any additional treatment.

The highest xylose concentration (12.5 g/L) was achieved for the saccharification of shiitake SMS (Figure 6A). The xylose formation in the saccharification of hydrothermally treated oyster mushroom SMS was comparable for partially isothermal (7.0 ± 0.2 g/L) and non-isothermal (6.3 ± 0.2 g/L) heating modes (*p* > 0.05). The significantly lower xylose formation for hydrothermally treated oyster mushroom SMS compared to shiitake SMS (*p* < 0.01) can be explained by the lower xylan content in the pretreated solids (Table 2) because of solubilization during treatment.

Most glucose and xylose were released during the first 24 hours (Figure 6A). For example, for the oyster mushroom pretreated under the partially isothermal mode, glucose concentration at the 24-hour point was 34.8 g/L, corresponding to 88% of the concentration achieved during the whole saccharification time. A similar trend was observed for both oyster mushroom SMS samples regarding glucose and xylose. The saccharification was even faster for shiitake SMS since ~ 85% of the produced glucose and xylose was already detected at the 10-hour point. Since the increase of sugar concentration observed between 24 h and 72 h for hydrothermally treated oyster SMS and between 10 h and 72 h for shiitake SMS was only marginal, it is evident that the saccharification duration can be considerably shortened.

To better assess the effect of the HT treatment, one must go beyond the mere determination of sugar concentration in the hydrolyzates, i.e. the percentage of saccharified polysaccharides out of the initial content should be determined using mass balances. The assessment can be based on the polysaccharides in the treated solids or the raw material before HT treatment. For the oyster mushroom SMS treated under partially isothermal heating, the enzymatic digestibility of treated cellulose (EDP-C) was 75.8% (w/w) (Figure 6B). That means 75.8% of the cellulose in the treated solids was saccharified. For the sample treated under non-isothermal heating mode, the EDP-C was considerably lower (38.9%), and a similar trend was observed for the enzymatic digestibility of treated xylan (EDP-X). This indicates that for oyster mushroom SMS, the HT treatment under partially isothermal mode was more effective than the non-isothermal one in enhancing the cellulose digestibility. However, one should remember that the biomass is held longer at the working temperature for the partially isothermal mode, which implies higher expenses. Although the values are lower than those achieved in the analytical enzymatic saccharification procedure (Figure 5), the trend is comparable. The dissimilarity can be explained by the geometric and dynamic differences and mass transfer variations between the experiments in the Eppendorf tubes and in the shake flasks, as well as by the different rheology caused by a larger solids’ consistency in the reaction system.

The assessment based on the initial cellulose revealed an overall enzymatic conversion (OEC-C) of 61.8% (w/w) for the oyster SMS pretreated under partially isothermal mode (Figure 6B). This means that 61.8% of the cellulose contained in the SMS before being submitted to HT treatment was saccharified. So, the cellulose losses that occurred during treatment are considered. The OEC-C was lower (32.4%) for the sample treated under non-isothermal mode. On the other hand, shiitake SMS resulted in an enzymatic conversion of 56.7%, comparable to the OEC-C calculated for the best-performing treated oyster mushroom SMS. For xylan, the trend was much more favorable for shiitake SMS than hydrothermally treated oyster SMS. The overall enzymatic conversion of xylan (OEC-X) was two-fold higher for shiitake SMS (55.7% (w/w)) than for both samples of treated oyster SMS (22.2–24.7% (w/w). These values are even more favorable considering that shiitake SMS does not require additional treatment, which means that the observed conversions are achieved at a lower cost.

### 3.7. Characterization of the SMS saccharification residue

After the saccharification of cellulose and hemicelluloses from SMS, a solid residue containing lignin and the non-saccharified portion of polysaccharides was generated. The amount of generated residue was in correspondence with the observed saccharification trends. For example, for the oyster SMS treated under partially isothermal conditions, which was the best-saccharified material, the generated residues corresponded to 32.4% of the dry mass of the initial SMS (Data not shown). For shiitake SMS and the oyster SMS sample treated non-isothermally, the generated residue corresponded to 35.2% and 50.2% of the initial dry mass, respectively.

The solid residues from the experimental replicates of the preparative enzymatic saccharification were pooled and submitted to compositional analysis. The results are presented in Table 3.

**Table 3.** Composition (% w/w) of the residues of the saccharification of spent mushroom substrates of shiitake and oyster mushroom. Mean values and standard deviations from triplicate measurements.

Component	Shiitake SMS	Oyster mushroom SMS
Cellulose^1^	20.8 ± 0.2	24.6 ± 1.0
Xylan	1.3 ± < 0.1	2.3 ± 0.2
Lignin^2^	24.7 ± 1.9	35.2 ± 1.0
Water extractives	25.7 ± 0.3	10.1 ± 0.2
Ethanol extractives	4.2 ± 0.1	1.2 ± 0.2
Ash	3.6 ± < 0.1	2.1 ± < 0.1

^1^Determined as glucan; ^2^Determined as Klason lignin.

Comparing the composition of the enzymatic saccharification residues with that of the initial SMS, it is evident that the saccharification residues are enriched in lignin and have a decreased content of polysaccharides. Lignin content, which was only 6.6–12.7% in the initial SMS (Table 1), increased to 28.1–30.6% (w/w) in the saccharification residues (Table 3). The increase is due to the saccharification of cellulose and hemicelluloses, which is also reflected in the decrease of the content of cellulose (from around 30% in the raw SMS to 20–25% in the saccharification residue), xylan (from 11–16% to 1–2%), and arabinan (from 2–3% to depletion). Extractive compounds also represented a substantial share of the mass of the saccharification residues, especially for shiitake SMS.

### 3.8. Lignin recovery from the SMS saccharification residue

The residue of the enzymatic saccharification of shiitake SMS was treated with the green solvent γ-valerolactone (GVL) to extract lignin. Five extraction conditions were assayed at temperatures between 120 and 150°C and between 20 and 40 min. The conditions were selected based on pilot experiments and previous experience with lignin extraction from the enzymatic saccharification residue of a spruce-based biorefinery process [26]. The current study is the first report on lignin extraction from SMS saccharification residue. There is one report on lignin extraction from SMS [22], but it focuses on raw SMS rather than on saccharification residue.

**Figure 7.**
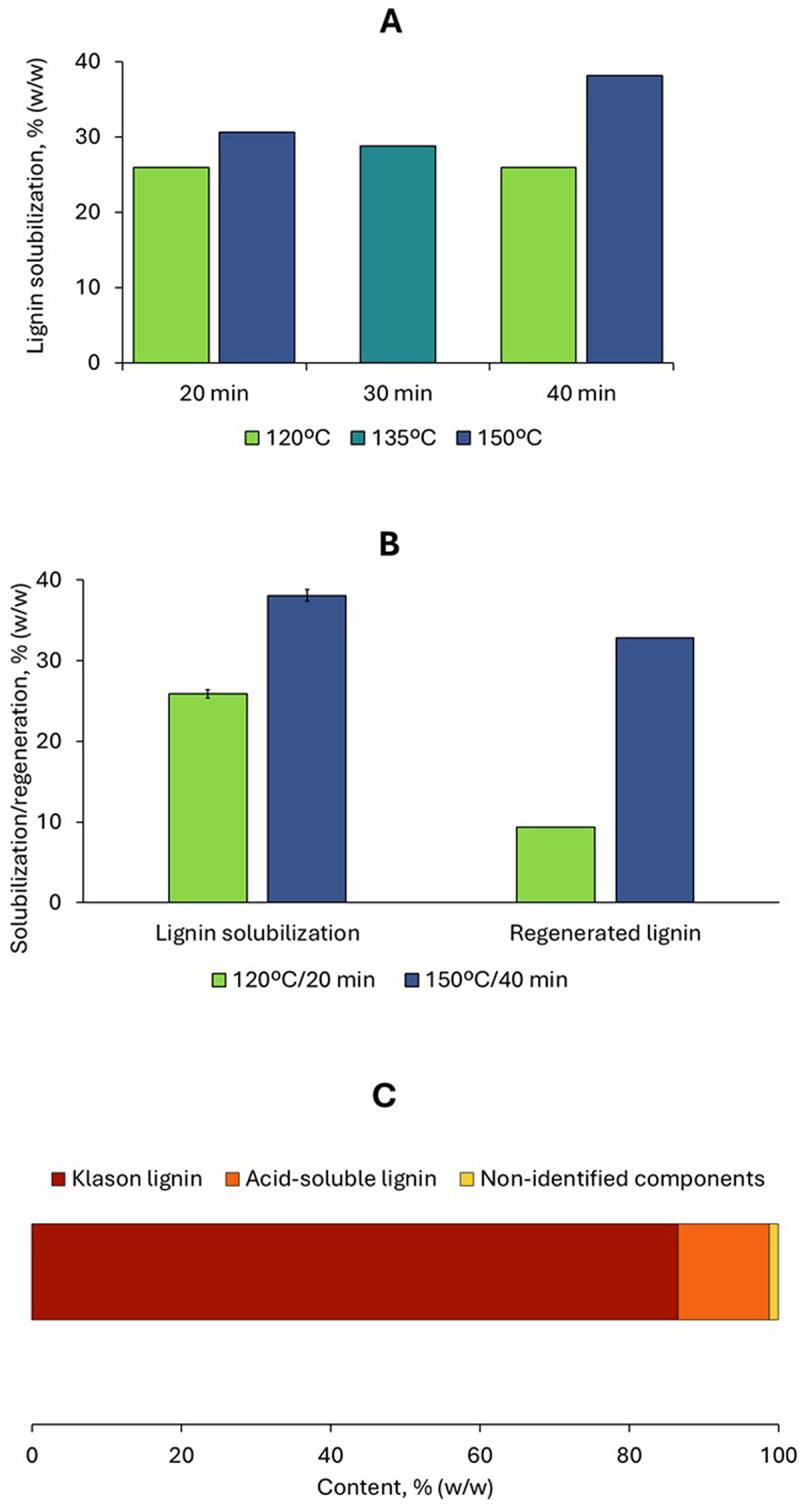
Lignin extraction from the saccharification residue of shiitake SMS using GVL. A, lignin solubilization from all the extraction conditions; B, comparison of lignin solubilization and regeneration for two selected conditions; C, composition of the regenerated lignin.

The GVL treatment led to the solubilization of 26–38% (w/w) of the lignin found in the saccharification residue (Figure 7A). The experiments carried out at 150°C resulted in higher lignin solubilization than those at 120°C. The highest value (38.1%) was achieved at 150°C for 40 min. The degree of solubilization is below the 50–64% solubilization we previously achieved in GVL extraction of spruce hydrolysis lignin [26] and steam-exploded spruce. The reason behind the difference is the lowest temperature (up to 150ºC) and shortest time (up to 40 min) in the current study compared with the conditions of the mentioned studies (up to 210°C and 2 h). In further studies, the operational conditions will be tuned to maximize the lignin extraction from SMS.

The anti-solvent procedure used for regenerating lignin from the liquors resulted in an apparently high precipitation. However, the separation process was not effective enough to achieve a high recovery of the precipitated lignin. A large part of the precipitated lignin remained in the supernatants or in the filtrates when separation by either centrifugation or filtration was attempted. Higher recovery of regenerated lignin was observed for the experiments at 150°C, where lignin solubilization was higher than for those at 120°C, where solubilization was lower (Figure 7B). The highest recovery (32.8% (w/w) of the solubilized lignin) was achieved for the extraction performed at 150°C for 40 min. The recovery trend is in agreement with the results of a previous study on GVL upgrading of spruce hydrolysis lignin, where lignin recovery was easier from conditions resulting in higher lignin solubilization than from systems with low solubilization yields [26]. The incomplete lignin regeneration after GVL extraction has also been reported for corn stalks [37] and eucalyptus chips [38]. This is the first report within a series of studies on lignin recovery from SMS saccharification residues, and it is logical to expect improvements as the method gets optimized. In further studies, lignin recovery will be potentiated by a sequential decrease of GVL concentration, as shown by Wang et al. [37], and by applying ultrasonic treatment [39].

Due to the low recovery of the regenerated lignin, the mass from each extraction condition was not enough for characterization. Therefore, the samples from all the experimental runs were pooled into a larger batch and then submitted to analytical acid hydrolysis for compositional analysis [29]. The analysis revealed that the regenerated lignin was composed of 86.6% (w/w) Klason lignin and 12.2% (w/w) acid-soluble lignin (Figure 7C). The regenerated lignin did not contain carbohydrates, considering that no monosaccharide signals were observed in the chromatograms of the analytical acid hydrolyzates. Around 1.2% of the lignin mass share was not identified. The high lignin content is comparable to the range of 89–92% previously reported by us for the product resulting after GVL extraction of spruce softwood lignin [26].

Even if the results of the lignin solubilization and regeneration are still sub-optimal, the high purity of the regenerated product reveals the potential of GVL for extracting lignin from the residues from SMS saccharification. This, together with the above-discussed results on an improved enzymatic digestibility of extract-free SMS, sets the ground for SMS valorization through a biorefinery process including extraction of bioactive compounds, production of fermentable sugars, and recovery of high-quality lignin for high-added value applications.

## 4. Conclusions

The results of this study demonstrate that the spent substrate from shiitake cultivation is more susceptible to enzymatic saccharification than that from oyster mushroom cultivation. This shows the potential of shiitake cultivation as a biological pretreatment method for bioconversion of lignocellulosic biomass.

The potential of hydrothermal treatment and γ-valerolactone extraction for applications in SMS biorefining was shown. Hydrothermal treatment under mild conditions effectively enhanced the enzymatic saccharification of cellulose in oyster mushroom SMS by over 84% compared to the untreated material, reaching levels comparable to those achieved for shiitake SMS. Even if the results are not optimal yet, the capability of γ-valerolactone extraction for enabling lignin recovery from the residues from SMS enzymatic saccharification was demonstrated.

The enhancement of the enzymatic digestibility of SMS polysaccharides after removal of the extractive compounds and the potential of the saccharification residue as a lignin source open the possibilities for a biorefinery process allowing recovery of bioactive compounds, fermentable sugars, and high-quality lignin from SMS.

## Data Availability

The authors confirm that the data backing these results are available within the article. After finishing the project, raw data will be deposited in the INN Open Research Data repository within DataverseNO. Detailed information can be made available upon reasonable request.

## Research Highlights

- The capability of shiitake and oyster mushrooms to pretreat biomass was compared
- Shiitake spent substrate was highly susceptible to enzymatic saccharification
- Mild treatment enhanced saccharification of oyster mushroom spent substrate by up to 84%
- γ-Valerolactone is used for extracting lignin from saccharification residues
- The potential of spent mushroom substrate as a biorefinery feedstock is shown

## References

[1] Atila F, Ogutcu H, Bilginoglu E, et al. Effect of phenolic-rich forest and agri-food wastes on yield, antioxidant, and antimicrobial activities of *Ganoderma lucidum*. Biomass Conv Bioref. 2024;14:25811–18. doi: 10.1007/s13399-023-04708-6

[2] Zhang Y, Geng W, Shen Y, et al. Edible mushroom cultivation for food security and rural development in China: bio-innovation, technological dissemination and marketing. Sustainability. 2014;6(5):2961–2973. doi: 10.3390/su6052961

[3] Beka D, Kenemi A. Effect of substrate composed from pea straw, wastepaper and wheat bran on growth and biological efficiency of oyster mushroom (*Pleurotus ostreatus*), in Ethiopia. Int J Agric Nat Sci. 2024;17(1):1–12.

[4] Zisopoulos FK, Ramírez HAB, van der GA, et al. A resource efficiency assessment of the industrial mushroom production chain: the influence of data variability. J Cleaner Production. 2016;126:394–408. doi: 10.1016/j.jclepro.2016.03.066

[5] Leong YK, Ma TW, Chang JS, et al. Recent advances and future directions on the valorization of spent mushroom substrate (SMS): a review. Bioresour Technol. 2022;344:126157. doi: 10.1016/j.biortech.2021.126157 34678450

[6] Mohd Hanafi FH, Rezania S, Mat Taib S, et al. Environmentally sustainable applications of agro-based spent mushroom substrate (SMS): an overview. J Mater Cycles Waste Manag. 2018;20(3):1383–1396. doi: 10.1007/s10163-018-0739-0

[7] Martin C, Zervakis GI, Xiong S, et al. Spent substrate from mushroom cultivation: exploitation potential towards various applications and value-added products. Bioengineered. 2023;14(1):2252138. doi: 10.1080/21655979.2023.2252138 37670430 PMC10484051

[8] Martín C, Xiong S, Passoth V, et al. Mushrooms for enhanced agriculture sustainability – The MUSA concept. C3-Bioecon: Circular Sustain Bioecon. 2023 4;(4):131–147. doi: 10.21071/c3b.vi4.16591

[9] Chen F, Martín C, Lestander TA, et al. Shiitake cultivation as biological preprocessing of lignocellulosic feedstocks – Substrate changes in crystallinity, syringyl/guaiacyl lignin and degradation-derived by-products. Bioresour Technol. 2022;344:126256. doi: 10.1016/j.biortech.2021.126256 34737055

[10] Chen F, Xiong S, Gandla ML, et al. Spent mushroom substrates for ethanol production – Effect of chemical and structural factors on enzymatic saccharification and ethanolic fermentation of *lentinula edodes*-pretreated hardwood. Bioresour Technol. 2022;347:126381. doi: 10.1016/j.biortech.2021.126381 34813922

[11] Paulsen Thoresen P, Lange H, Crestini C, et al. Characterization of organosolv birch lignins: toward application-specific lignin production. ACS Omega. 2021;6(6):4374–4385. doi: 10.1021/acsomega.0c05719 33623848 PMC7893791

[12] Klausen SJ, Falck-Ytter AB, Strætkvern KO, et al. Evaluation of the extraction of bioactive compounds and the saccharification of cellulose as a route for the valorization of spent mushroom substrate. Molecules. 2023;28(13):5140. doi: 10.3390/molecules28135140 37446802 PMC10343705

[13] Gandla ML, Tang C, Martín C, et al. Enzymatic saccharification of lignocellulosic biomass. In: Jeschke P, and Starikov EB. eds. In Agricultural Biocatalysis: Enzymes in Agriculture and Industry. Singapur: Jenny Stanford Publishing; 2022. p. s. 413–69.

[14] Xiong S, Martín C, Eilertsen L, et al. Energy-efficient substrate pasteurisation for combined production of shiitake mushroom (*Lentinula edodes*) and bioethanol. Bioresour Technol. 2019;274:65–72. doi: 10.1016/j.biortech.2018.11.071 30500765

[15] Jönsson LJ, Martín C. Pretreatment of lignocellulose: formation of inhibitory by-products and strategies for minimizing their effects. Bioresour Technol. 2016;199:103–112. doi: 10.1016/j.biortech.2015.10.009 26482946

[16] Kuhad RC, Rapoport A, Kumar V, et al. Biological pretreatment of lignocellulosic biomass: an environment-benign and sustainable approach for conversion of solid waste into value-added products. Crit Rev Environ Sci Technol. 2023;54(10):771–796. doi: 10.1080/10643389.2023.2277670

[17] Chen F, Xiong S, Sundelin J, et al. Potential for combined production of food and biofuel: cultivation of *Pleurotus pulmonarius* on soft- and hardwood sawdusts. J Cleaner Production. 2020;266:122011. doi: 10.1016/j.jclepro.2020.122011

[18] Kumla J, Suwannarach N, Sujarit K, et al. Cultivation of mushrooms and their lignocellulolytic enzyme production through the utilization of agro-industrial waste. Molecules. 2020;25(12):2811. doi: 10.3390/molecules25122811 32570772 PMC7355594

[19] Chen F, Martín C, Finell M, et al. Enabling efficient bioconversion of birch biomass by *Lentinula edodes*: regulatory roles of nitrogen and bark additions on mushroom production and cellulose saccharification. Biomass Conv Bioref. 2022;12(4):1217–1227. doi: 10.1007/s13399-020-00794-y

[20] de Cássia Spacki K, Novi DMP, de oliveira-Junior VA, et al. Improving enzymatic saccharification of peach palm (*Bactris gasipaes*) wastes via biological pretreatment with *pleurotus ostreatus*. Plants. 2023;12(15):2824. doi: 10.3390/plants12152824 37570978 PMC10420912

[21] Zhu Y, Chang Y, Guan J, et al. Butanol production from organosolv treated spent mushroom substrate integrated with in situ biodiesel extraction. Renewable Energy. 2016;96:656–661. doi: 10.1016/j.renene.2016.04.048

[22] Beckers SJ, Dallo IA, Del Campo I, et al. From compost to colloids—valorization of spent mushroom substrate. ACS Sustain Chem Eng. 2019;7(7):6991–6998. doi: 10.1021/acssuschemeng.8b06710

[23] Ghose TK. Measurement of cellulase activities. Pure Appl Chem. 1987;59(2):257–268. doi: 10.1351/pac198759020257

[24] Ghose T, Bisaria V. Measurement of hemicellulase activities part 1: Xylanases. Pure Appl Chem. 1987;59(12):1739–1752. doi: 10.1351/pac198759121739

[25] Liaqat A. Assessment of the enzymatic saccharification of polysaccharides contained in spent mushroom substrate; 2024 [MSc thesis]. Inland Norway University. cited 2024 Dec 24th. Available from: https://hdl.handle.net/11250/3139482

[26] Momayez F, Hedenström M, Stagge S, et al. Valorization of hydrolysis lignin from a spruce-based biorefinery by applying γ-valerolactone treatment. Bioresour Technol. 2022;359:127466. doi: 10.1016/j.biortech.2022.127466 35710049

[27] Sluiter A, Ruiz R, Scarlata C, et al. Determination of extractives in biomass: laboratory analytical procedure (LAP). Technical report NREL/TP-510–42619. National Renewable Energy Laboratory; 2005.

[28] Sluiter A, Hames R, Ruiz C, et al. Determination of ash in biomass: laboratory analytical procedure (LAP). Technical report NREL/TP-510–42622. National Renewable Energy Laboratory; 2005.

[29] Sluiter A, Hames R, Ruiz C, et al. Determination of structural carbohydrates and lignin in biomass: laboratory analytical procedure (LAP). Technical report NREL/TP-510–42618. National Renewable Energy Laboratory; 2008.

[30] Atila F. Compositional changes in lignocellulosic content of some agro-wastes during the production cycle of shiitake mushroom. Scientia Hortic. 2019;245:263–268. doi: 10.1016/j.scienta.2018.10.029

[31] Li X, Pang Y, Zhang R. Compositional changes of cottonseed hull substrate during P. ostreatus growth and the effects on the feeding value of the spent substrate. Bioresour Technol. 2001;80(2):157–161. doi: 10.1016/S0960-8524(00)00170-X 11563708

[32] Gandla ML, Martín C, Jönsson LJ. Analytical enzymatic saccharification of lignocellulosic biomass for conversion to biofuels and bio-based chemicals. Energies. 2018;11(11):2936. doi: 10.3390/en11112936

[33] Paz-Cedeno FR, Carceller JM, Iborra S, et al. Stability of the Cellic CTec2 enzymatic preparation immobilized onto magnetic graphene oxide: assessment of hydrolysis of pretreated sugarcane bagasse. Ind Crops Products. 2022;183:114972. doi: 10.1016/j.indcrop.2022.114972

[34] Müller G, Chylenski P, Bissaro B, et al. The impact of hydrogen peroxide supply on LPMO activity and overall saccharification efficiency of a commercial cellulase cocktail. Biotechnol Biofuels. 2018;11(1):209. doi: 10.1186/s13068-018-1199-4 30061931 PMC6058378

[35] Oliva-Taravilla A, Carrasco C, Jönsson LJ, et al. Effects of biosurfactants on enzymatic saccharification and fermentation of pretreated softwood. Molecules. 2020;25(16):3559. doi: 10.3390/molecules25163559 32764287 PMC7465028

[36] Martín C, Dixit P, Momayez F, et al. Hydrothermal pretreatment of lignocellulosic feedstocks to facilitate biochemical conversion. Front Bioeng Biotechnol. 2022;10:10. doi: 10.3389/fbioe.2022.846592 PMC8888528 35252154

[37] Wang G, Liu X, Yang B, et al. Using green γ-valerolactone/water solvent to decrease lignin heterogeneity by gradient precipitation. ACS Sustain Chem Eng. 2019;7(11):10112–10120. doi: 10.1021/acssuschemeng.9b01641

[38] Lê HQ, Ma Y, Borrega M, et al. Wood biorefinery based on γ-valerolactone/water fractionation. Green Chem. 2016;18(20):5466–5476. doi: 10.1039/C6GC01692H

[39] Li SX, Li MF, Yu P, et al. Valorization of bamboo by γ-valerolactone/acid/water to produce digestible cellulose, degraded sugars and lignin. Bioresour Technol. 2017;230:90–96. doi: 10.1016/j.biortech.2017.01.041 28161625

